# Celiac Disease: A Common Unrecognized Health Problem with a Very Delayed Diagnosis

**DOI:** 10.3390/medicina56010009

**Published:** 2019-12-26

**Authors:** Luis Rodrigo

**Affiliations:** Gastroenterology Unit, Hospital Universitario Central de Asturias, 33011 Oviedo, Asturias, Spain; lrodrigosaez@gmail.com

Celiac disease (CD) is a clinical entity of autoimmune nature, related to the presence of a permanent gluten intolerance that affects genetically predisposed individuals, producing a chronic inflammation process that usually occurs in the small bowel. It is accompanied by a relatively high frequency of simultaneous or successive involvement of various extra-digestive organs over time [1].

CD has an extensive epidemiological distribution, affecting all countries and ethnicities. Its average prevalence is 1–2% in the general population, with slight variations between geographical areas. It can arise at any time during the life-course, but predominantly appears in middle age, and up to 20% of cases are diagnosed in patients older than 60 years. It is also clearly predominant in women (average ratio 2:1, female:male) [2,3].

Clinical manifestations vary considerably in relation to the age of presentation and to various associated exogenous factors. In children, it usually begins to manifest itself in conjunction with the introduction into their diet of foodstuffs containing wheat flour (e.g., porridge) from six months of age. In the most severe cases, the clinical symptoms appear before the age of two years. In general, digestive symptoms predominate, such as chronic diarrhea, bloating and weight loss, the “classic triad” of symptoms, and are not necessarily accompanied by a malabsorption syndrome. Other accompanying symptoms are anorexia, vomiting, reflux and accentuated irritability, along with episodes of constipation that can be frequent and prolonged [4,5].

When the disease appears in older children or adolescents, several extraintestinal manifestations, such as headaches, arthritis, anemia and accentuated asthenia, among others, may appear in addition to digestive symptoms [6].

The forms of CD presentation are very varied in adults, with frequent associations of intestinal and extradigestive symptoms, often referred to as “atypical forms”. Among them are serious conditions, such as chronic anemia, osteoporosis, a variety of skin lesions, polyneuritis, migraines, persistent liver test abnormalities, dysmenorrhea, amenorrhea, fertility disorders, recurrent abortions and mood changes (e.g., irritability and depression). Dermatitis herpetiformis is the skin lesion most frequently associated with CD, appearing in up to 25% of cases. It is easily recognized and highly suspicious. Gluten is the agent mainly responsible for the condition, and its withdrawal is definitively the most effective treatment.

Initial clinical screening most often involves determining levels of serological markers, which are circulating antibodies directed against some compound of gluten proteins, sensitivity or the enzymes that metabolize it. The most commonly used are class 2 anti-tissue transglutaminase antibodies, which are close to 90% efficacious in cases with intestinal villus atrophy. However, their diagnostic sensitivity is remarkably low, at 30–40%, for cases without villous atrophy, so that one or more negative determinations does not in any way rule out the possibility that an established CD is present [7].

There are two known genetic markers, both belonging to the HLA-II class, available for routine clinical use in the study of patients with CD. HLA-DQ2 is the most frequent, being positive in 90% of celiac patients, while HLA-DQ8 is much less common (5–8%). The two genetic markers are simultaneously negative in a small percentage of patients (<2–3%). The presence of both genetic markers is considered to be a necessary, but not sufficient condition for the diagnosis, as they also occur in up to 30% of the general non-celiac population.

The spectrum of duodenal histological changes in CD has expanded greatly since the inclusion of the new criteria introduced by Marsh in 1992 [8]. He successfully included celiac patients without villous atrophy, classifying them as type 1 when there was only an increased intraepithelial lymphocytosis (LIES) (>25% of LIES, per 100 epithelial cells). Type 2 is characterized by the presence of crypt hyperplasia without atrophy. Type 3, showing villous atrophy, is subdivided into three categories: mild (3a), moderate (3b) and intense (3c). Other classifications have since appeared, but they are basically very similar to the original Marsh classification [9,10].

The most important step towards achieving a diagnosis of CD is that every doctor looks for this entity and includes it in their differential process before a series of symptoms, and not only digestive but also long-term extra-intestinal usually. This is not achieved solely on the basis of clinical data and exploratory findings, but aided by analytical alterations, serological data, genetic markers and duodenal histopathological findings. If, after this process, reasonable doubts remain about its presence, it may be tentatively proposed that the patient follows a gluten-free diet (GFD) for at least six months, to assess their degree of response. Although a GFD is the only available and effective treatment, it should be made clear that it must be followed strictly and maintained for the rest of the patient’s life, avoiding transgressions and contamination [11].

Diagnosis is often delayed, the time following symptom onset being highly variable in adults, sometimes taking as long as 12 years. Barriers to accurate and timely diagnosis include atypical presentation, physicians’ lack of awareness about current diagnostic criteria, misdiagnosis and general practitioners’ limited access to specialists [12]. In a survey of 611 CD patients in Finland, 332 (54%) reported a delay in diagnosis of more than three years. This delay predisposed patients to reduced well-being and increased recourse to medicines and health care services, before the diagnosis and one year after diagnosis [13]. New guidelines have been issued for children who exhibit high serum TGT titers of more than ten times the normal value. In such cases it is not considered necessary to perform duodenal biopsies to confirm the diagnosis [14].
